# The influence of *Echinacea purpurea* leaf microbiota on chicoric acid level

**DOI:** 10.1038/s41598-019-47329-8

**Published:** 2019-07-26

**Authors:** Valentina Maggini, Marinella De Leo, Carlotta Granchi, Tiziano Tuccinardi, Alessio Mengoni, Eugenia Rosaria Gallo, Sauro Biffi, Renato Fani, Luisa Pistelli, Fabio Firenzuoli, Patrizia Bogani

**Affiliations:** 1 Department of Biology, University of Florence, Via Madonna del Piano 6, 50019 Sesto Fiorentino, Italy; 2 Department of Experimental and Clinical Medicine, University of Florence, Largo Brambilla 3, 50134 Florence, Italy; 3 Referring Center for Phytotherapy, Tuscany Region, Careggi University Hospital, Largo Brambilla 3, 50134 Florence, Italy; 4 Department of Pharmacy, University of Pisa, Via Bonanno 6 and 33, 56126 Pisa, Italy; 5 Research Centre for Nutraceutical and Healthy Foods “NUTRAFOOD”, University of Pisa, via del Borghetto 80, 56124 Pisa, Italy; 6 Botanical Garden Casola Valsenio, Via del Corso 6, 48010 Ravenna, Italy

**Keywords:** Microbiome, Secondary metabolism

## Abstract

The controversial anti-proliferative effects of *Echinacea purpurea* (L.) Moench (Asteraceae) might be related to different plant metabolites contained in plant samples, extracts and products. The influence of bacterial endophytes on the synthesis of bioactive compounds in the medicinal plants has been previously demonstrated but there are only few studies addressing anticancer effects and mechanisms of *E*. *purpurea* extracts following endophytic colonization. The present study aimed to test and compare the lactate dehydrogenase (LDH) inhibition potential of *n*-hexane and methanol extracts from *in vitro* endophyte non-inoculated and inoculated *E*. *purpurea* plants. An *in vitro* model was previously set up to perform the infection of axenic *E*. *purpurea* plants with bacterial endophytic strains isolated from *E*. *purpurea* aerial part. Only methanol extracts showed LDH5 inhibition, in particular the richest in chicoric acid and most strongly inhibiting extract was obtained from inoculated stem and leaves of *E*. *purpurea* (IC_50_ = 0.9 mg/ml). Chicoric acid showed an IC_50_ value (66.7 µM) in enzymatic assays better than that of the reference compound galloflavin. Modeling studies were carried out to suggest the putative interaction mode of chicoric acid in the enzyme active site. This *in vitro* model on plant-bacterial interaction may lead to obtain extracts from plants enriched in bioactive compounds and it is a new approach for the discovery of novel anticancer compounds.

## Introduction

*Echinacea purpurea* (L.) Moench (Asteraceae) is cultivated for the preparation of several best-selling herbal supplements^1^. A meta-analysis of randomized controlled trials has highlighted that *Echinacea* extracts are able to reduce the onset of recurrent respiratory infections and complications^2^. Moreover, preclinical studies have also indicated immune-modulatory and anti-inflammatory properties of *E*. *purpurea* extracts^3^ and conflicting results about their potential effort to cancer treatment have been reported^1^. Since the biological activity of *Echinacea* spp. may be influenced by secondary metabolites such as alkylamides, caffeic acid, and chlorogenic acid^4^, the controversial anti-proliferative effects could be related to different metabolite (*e*.*g*. alkamides and chicoric acid) concentrations. These differences are due to various factors, including plant organs, climatic factors, growing conditions, water availability and storage^3^. Endophytic fungi of the medicinal plants have been also extensively studied^5,6^ showing that they can produce analogues of plant molecules^7,8^. In the last few years, remarkable attention also focused on the influence of bacterial endophytes on the synthesis of bioactive compounds in the medicinal plants^9,10^. It has been previously demonstrated that the production of *Echinacea* bioactive compounds is influenced by the plant microbiota^11^. Axenic *in vitro E*. *purpurea* plants were inoculated with a consortium of bacterial strains isolated from the *E*. *purpurea* aerial parts (stems and leaves) to set up a model able to display that the endophytes can influence the alkamide levels in plant compartments. In particular, the *n*-hexane extracts of inoculated *E*. *purpurea* tissues have shown a higher level of alkamides than the extracts of control (non-inoculated) plants. Then, we have been intrigued by the possibility to test and compare the anticancer activity of these extracts. Since chicoric acid has shown anti-proliferative properties^1^, also methanol extracts (mainly containing chicoric acid) of control and inoculated *E*. *purpurea* tissues have been considered. The extracts were tested for their lactate dehydrogenase (LDH) inhibitory potency. In fact, the human isoform lactate dehydrogenase 5 is an enzyme overexpressed in many invasive tumours and it represents a challenging anti-cancer target. LDH5 covers a key role for what concerns the energetic metabolism of tumours, since it catalyses the last step of the glycolytic process, which is the reduction of pyruvate to lactate, therefore allowing cancer cells to produce ATP by aerobic glycolysis even when they are in the presence of low oxygen concentrations. Therefore, LDH5 inhibition should promote cancer cell death by starvation, maintaining healthy cells alive, since they usually rely of oxidative phosphorylation to produce ATP. Many synthetic LDH5 inhibitors have been reported, and in the last years a growing number of natural compounds has shown promising activities on this target^12,13^, thus prompting the present investigation.

The choice of LDH as a putative target of *E*. *purpurea* extracts derived from the observation of the chemical structures of the main isolated compounds. The polar phenolic metabolites isolated from methanol extracts could nicely correspond to the chemical requirements of this enzyme, since most LDH5 inhibitors reported in literature possess carboxylic acids or hydroxyl groups in their structures^14^. Thus, we tested methanol and *n*-hexane extracts of *E*. *purpurea* and, after the first biological evaluation, the main component of the promising methanol extract, chicoric acid, was tested on LDH5.

## Results and Discussion

### Bacterial colonization in the *in vitro E*. *purpurea* model

In Supplementary Table S1 were reported the thirty-seven bacterial strains isolated from the *E*. *purpurea* aerial part and used to perform the bacterial infection of *E*. *purpurea in vitro* plants as described in Maggini *et al*.^11^. Plant bacterial colonization was estimated forty-five days after the infection by the total viable count (TVC) as Colony Forming Units (CFU)/g resulting lower into the host roots than in the aerial parts^11^.

### Profiling analysis of phenolic content in different organs of control and inoculated plants

**Figure 1:**
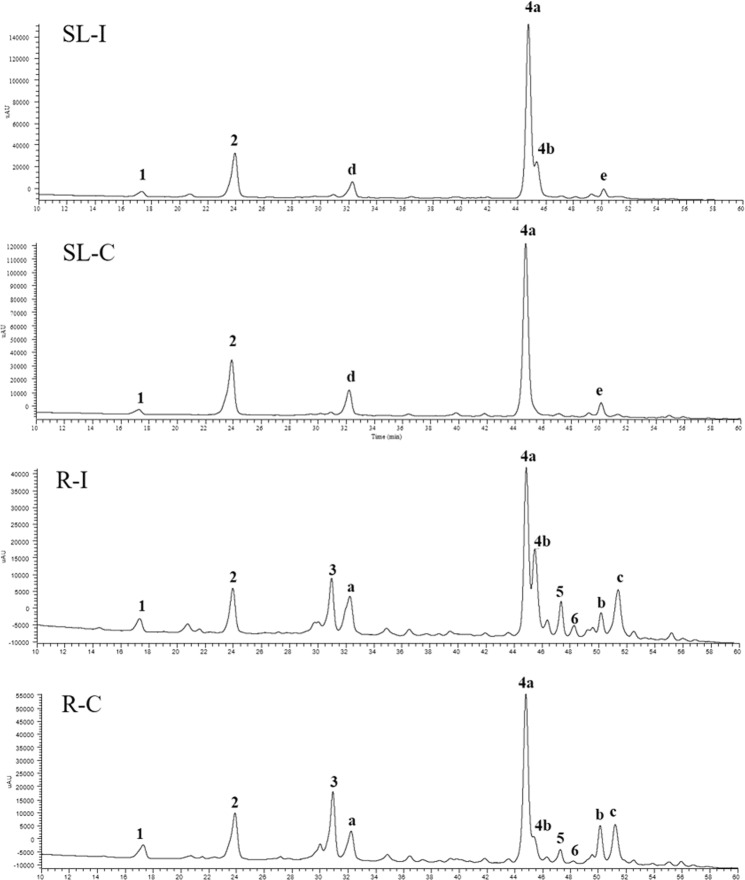
HPLC-PDA/UV profiles of methanol extracts of control roots (R-C) and aerial part (stem and leaves; SL-C) and endophyte-inoculated roots (R-I) and aerial part (stem and leaves; SL-I) of *Echinacea purpurea*, recorded at 330 nm. **1** = Dihydroxybenzoic acid hexoside; **2** = Caftaric acid; **3** = Chlorogenic acid; **4a**/**4b** = Chicoric acid; **5** = Dicaffeolylquinic acid; **6** = Rutin. Data of peaks **1**–**6** are described in Supplementary Table S2. Compounds **a**–**e** remained unidentified.

The chemical analyses of aerial part and root methanol extracts obtained from axenic and endophyte-inoculated plants by High Performance Liquid Chromatography (HPLC) coupled with Photo Diode Array/Ultraviolet (PDA/UV) and Electrospray Ionization tandem Mass Spectrometry (ESI-MS/MS) techniques were focused on the profile of phenolic constituents, particularly with regard to chicoric acid, that is known to be the main phenolic compound in both aerial parts and roots of *E*. *purpurea*^4^. Results of HPLC-PDA/UV analyses of all extracts, recorded at 330 nm, are shown in Fig. 1. The identification of all compounds was carried out by comparison of UV data, fragmentation patterns obtained from ESI-MS/MS experiments and elution order of detected molecules with those reported in the literature (Supplementary Table S2). In total, six phenolic acids were identified as a dihydroxybenzoic acid hexoside (**1**), caftaric acid (**2**), chlorogenic acid (**3**), chicoric acid isomers (**4a** and **4b**), and dicaffeoylquinic acid (**5**). However, a flavonol glycoside was identified in the roots as rutin (**6**). Compounds **3**, **4a**, and **6** were confirmed by injection in the LC-PDA/UV-ESI-MS system of authentic standards. Some peaks (compounds **a**-**e**) remained unidentified.

*E*. *purpurea* roots and aerial parts, both axenic and inoculated, displayed a different phenolic content, with three phenolic constituents (**1**, **2**, **4a**, and **4b**) detected in aerial parts and six (**1**–**6**) in roots. Components **1**, **2**, **4a**, and **4b** were detected in both aerial parts and roots organs, whereas constituents **3**, **5** and **6** were completely lacked in aerial parts. The most representative metabolite in all samples was compound **4a** (***t***_**R**_ = 44.8 minutes) identified as chicoric acid (2-caffeoyltartaric acid), based on its characteristic absorbances at 250 and 330 nm and the fragmentation pattern of deprotonated molecule [M-H]^−^ at *m/z* 473 showing ion products at *m/z* 311 ([M-H-162]^−^), due to the loss of a caffeoyl moiety, and 179 ([M-H-162-132]^−^), due to the subsequent loss of tartaric portion^15^. The injection of a standard chicoric acid confirmed these findings. In all analysed extracts, with the exception of control aerial part extract, the elution of an isomeric form was observed immediately after l-chicoric acid (**4b**, ***t***_**R**_ = 45.5 minutes). From the data available in the literature, a *meso*-form of chicoric acid was previously reported and the occurring in natural sources of other phenolic derivatives conjugated to a *meso*-tartaric acid could be possible, but it is still unclear whether the isomerization occurs under the extraction process^16^. Another caffeic ester of tartaric acid was compound **2** (***t***_**R**_ = 24.0 minutes, λ_max_ 255, 290, 330 nm), having molecular weight of 312 amu, as deduced by deprotonated molecular ion peak [M-H]^−^ at *m/z* 311 in the mass spectrum, and a structure characterized by a caffeoyl moiety and a tartaric unit, as shown by corresponding ion fragments at *m/z* 179 ([M-H-132]^−^) and 149 ([M-H-162]^−^) in the MS/MS experiments. Thus, compound **2** was identified as caftaric acid^15^. Compound **3**, detected at ***t***_**R**_ = 31.0 minutes, exhibited a λ_max_ at 245 and 330 nm. In the full mass spectrum a molecular ion peak [M-H]^−^ at *m/z* 353 was observed, while MS/MS displayed diagnostic peaks at *m/z* 191 and 179, corresponding to deprotonated quinic and caffeic acid, respectively, leading to identify **3** as a caffeoylquinic acid^17^. The caffeoyl moiety location on C-3 of quinic acid was established by comparison of chromatographic data with an authentic standard, thus compound **3** was finally established to be chlorogenic acid. The presence of chlorogenic acid was previously reported in *E*. *purpurea* roots^18^. Full mass spectrum of compound **5** (*t*_R_ = 46.3 minutes, λ_max_ 255, 285, 330 nm) showed a molecular ion peak [M-H]^−^ at *m/z* 515, while in the MS/MS a base peak *m/z* 353 ([M-162]^−^) and ion products at *m/z* 191 ([M-162-162]^−^), and 179 ([M-162-174]^−^), generated by the losses of one caffeoyl, two caffeoyl, and one caffeoyl plus one quinoyl residues, respectively, were observed, suggesting a dicaffeoylquinic acid structure for this molecule. Previous studies reported the presence of 1,3-dicaffeoylquinic acid (cynarin) as typical constituent of *E*. *angustifolia* roots, useful to discriminate *E*. *angustifolia* from *E*. *pallida*^19^. Indeed, in several reports cynarin was lacking in roots and aerial parts of *E*. *purpurea*^20,21^. On the contrary, cynarin was detected in plants and in commercial products of *E*. *purpurea*^22,23^. Even though the MS fragmentation pathway of compound **3** is superimposable to that of cynarin^22^, its retention time is not in accordance with previous studies, since in the elution order of phenolics detected in *Echinacea* species reverse phase HPLC analysis on C-18 columns is as follows: caftaric acid, chlorogenic acid, cynarin, and chicoric acid^20–22^. This evidence suggested that compound **3** was a cynarin isomer in which the position of caffeoyl groups can’t be deduced based on UV and MS data. In the HPLC analysis of artichoke samples, cynarin is more polar than isomers 3,4-, 3,5-, 1,5-, and 4,5-dicaffeoylquinic acids^24^, according to our results that showed compound **3** eluting much later. Compound **6** (*t*_R_ = 49.2 minutes), exhibited two intense absorption peaks at 260 and 355 nm, suggesting the presence of a flavonoid with a molecular weight of 610 amu, as deduced from full ESI mass spectrum ([M-H]^−^ at *m/z* 609). ESI-MS/MS experiments evidenced a base peak at *m/z* 301 ([M-H-162-146]^−^), corresponding to the deprotonated aglycon quercetin after loss of a disaccharide chain formed by one hexose and one deoxyhexose units. The product ion at *m/z* 463 ([M-H-146]^−^) led to suppose the presence of a rutinose sugar, constituted by one rhamnose and one glucose, thus compound **6** was identified as rutin, according to data reported in the literature^17^ and chromatographic characteristic of a reference standard. Rutin was previously detected in *E*. *purpurea* in roots^25^.

In addition to these identified compounds, other molecule structures remained not completely assigned. Compound **1**, detected both in control and inoculated roots and aerial parts, was characterized by a molecular ion peak at *m/z* 315 ([M-H]^−^) and a product ion at *m/z* 153, due to the neutral loss of 162 amu, ascribable to a hexose moiety from a dihydroxybenzoyl residue. These evidences are according with a dihydroxybenzoic acid hexoside. A compound with the same molecular weight, the hydroxytyrosol hexoside, was reported in *E*. *angustifolia;* however, its mass fragmentation pattern is characterized by a product ion at *m/z* 123 ([M-H-162-30]^−^), whereas in the MS/MS spectra of compound **1** a fragment at *m/z* 109, due to the subsequent losses of an hexose and a carboxyl group ([M-H-162-44]^−^), can be observed, supporting the hypothesis of a dihydroxybenzoic acid as aglycone^26^. Considering other major peaks, such as compounds **a**-**e**, the identification was not possible based on chromatographic and spectral data. Peak **a** and **e**, showed molecular ions [M + HCOO]^−^ at *m/z* 427 and 591, respectively, whose fragmentation patterns were not informative for the structural characterization. Peak **b**, **c**, and **d** were generated by a mixture of coeluting compounds, thus difficult to identify.

Results from estimation of hydroxycinnamic derivative amount in all *E*. *purpurea* samples are listed in Supplementary Table S3. Our data indicated that endophyte-inoculated aerial parts are the richest in hydroxycinnamic derivatives (0.64 mg/g of fresh weight), followed by non-inoculated roots (0.51 mg/g of fresh weight). The axenic aerial parts showed less hydroxycinnamic derivative amount (0.48 mg/g of fresh weight) compared to the inoculated counterpart, whereas axenic roots contained an amount of hydroxycinnamic derivatives that was about twice compared to inoculated roots (0.29 mg/g of fresh weight).

**Figure 2:**
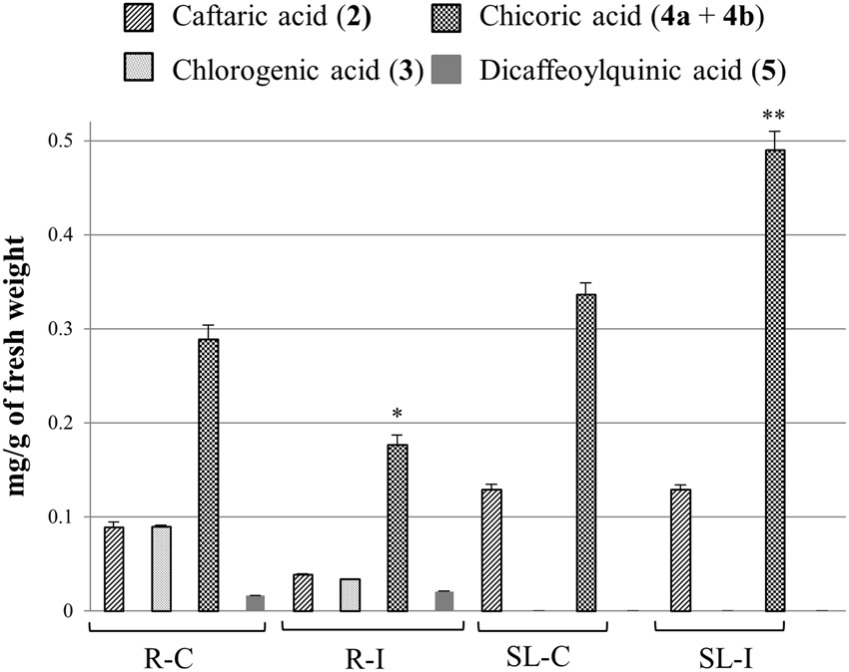
Influence of endophyte infection on phenolic level in *Echinacea purpurea* tissues. R-C = control roots, R-I = endophyte-inoculated roots; SL-C = control aerial part (stem and leaves); SL-I = endophyte-inoculated aerial part (stem and leaves). Compound numbers correspond with peak numbers in Fig. 1 and Supplementary Table S2. *P < 0.05, **P < 0.01 versus the relative control group.

A Principal Component Analysis (PCA, Supplementary Fig. S1) was conducted: the vectors computing data of the aerial parts (SL) extracts were differently directed than those of the roots (R) (F = 17.85; *P* < 0.001). Aerial parts presented chicoric acid isomers (**4a + 4b**) and caftaric acid (**2**), whilst the roots mainly contained chlorogenic acid (**3**). The comparison of mean phenolic contents (Supplementary Table S3) proved that the amount of chicoric acid isomers was significantly different between control aerial part (SL-C) and inoculated (SL-I) samples (Tukey HSD *P = *0.01). In fact, chicoric acid is the most abundant phenolic in all compartments and its content, calculated considering the two isomeric forms detected, is higher (about 30%) in inoculated aerial parts respect to the controls. Notably, in aerial parts the increment of total phenolic content is especially due to an increased level of chicoric acid isomers, whereas in the roots the infection produces a minor production of metabolites **1**–**4**, and a small increase of compounds **5** and **6** level (Fig. 2 and Supplementary Table S3).

Thus, the estimated total phenolic level was comparable between control roots and aerial parts in line with the current literature^27^. Interestingly, the highest and the lowest phenolic content was relatively found in inoculated aerial parts and roots, especially due to an altered level of chicoric acid isomers suggesting that it was influenced by the *E*. *purpurea* endophyte infection. Since bacterial communities are different between *E*. *purpurea* organs^28^ and the endophytes tended to newly colonize the native (aerial part) niche when inoculated in axenic plants^11^, aerial part endophytes could influence specific properties (*i*.*e*. increased chicoric acid level) in the leaves and not in the roots. In any case, stress due to plant-bacteria interaction was reported to generate phenolic accumulation^16^ but decreased chicoric acid levels following phytoplasma and virus infection were shown in *E*. *purpurea* roots^29^. Also, *E*. *purpurea* shoots displayed similar chicoric acid levels before and after arbuscular mycorrhizal fungus colonization whilst the roots resulted in increased chicoric acid^16^. These results suggested a differential influence of endophyte infection depending on plant and microorganism species and plant organ. Elicitors of biotic (yeast, fungi) or abiotic (synthetic organic compounds) origin have been reported^30,31^. Increased amounts of chicoric acid were also induced in *E*. *purpurea* by other elicitors (not of bacterial origin) as foliar application of fungal carbomethyl chitin glucan^32^, salicylic acid or novel titanium based molecules^31^. On the other hand, we showed that bacterial endophytes, as an elicitor, increased not only the amount of phenolics (present work) but also of alkamides^11^ pointing to obtain an augmented phytocomplex (extracted from plants enriched in different synergic bioactive compounds) able to carry out different physiological functions. Moreover, the endophytes naturally colonize the plant hosts acting as elicitors with no harmful or toxic effects. Also, endophytic bacteria could produce themselves medicinal compounds similar to those produced by the plant as reported for fungal endophytes^8^. In particular, chicoric acid biosynthesis is via the shikimic acid pathway also common to fungi and bacteria^33^. Thus, the endophytic bacterial consortia used in this work could produce similar analogs.

### Lactate dehydrogenase inhibition

Plant extracts were tested on human LDH5 isoform, with the aim of evaluating their inhibition potencies. The inhibitory activities of *n*-hexane and methanol extracts are reported as IC_50_ values (Table 1). Differently from the *n*-hexane extracts that proved to be inactive on LDH5, some of the methanol extracts showed promising inhibition potencies on the enzyme as reported for other phenolic compounds^34^. Control aerial part extracts displayed an IC_50_ value of 2.1 mg/ml and it was less potent that the inoculated counterpart, that reached the best IC_50_ value (0.9 mg/ml) among all the tested extracts, thus being even more potent that the corresponding inoculated root extract (IC_50_ = 1.9 mg/ml). In agreement with the trend observed in the inhibition activities of stem/leaves extracts, control root fraction was very less active than inoculated roots, without reaching a measurable inhibition value at the tested concentration. This preliminary evaluation led us to test in enzymatic assays the main component of methanol extracts, chicoric acid **4a**. Chicoric acid showed an IC_50_ value of 66.7 µM, therefore it resulted to be more active than the reference LDH5 inhibitor galloflavin^35^. This result would explain why methanol extracts proved to be able to inhibit LDH5, due to their significant content of the metabolite chicoric acid. By comparing results concerning both the chemical profiling analysis and the LDH5 inhibition data, it could be observed that both inoculated root and aerial part extracts (low and high chicoric acid content, respectively) showed higher LDH5 inhibition respect to the relative control samples. Indeed, on the basis of the chicoric acid content, the activity of the inoculated roots was higher than the expected one: this result could be explained considering that the effect of the R-I extracts might be due to the (increased) biosynthesis of other compounds induced by endophyte inoculation. On the other hand, the inoculated aerial part extracts, the richest in chicoric acid, revealed the highest activity among all the tested samples.

**Table 1:** Human LDH5 inhibition potencies.

		LDH5^a^ IC_50_ (mg/ml)	LDH5^a^ IC_50_ (µM)
*n*-hexane extracts	R-I	>10.0	—
	R-C	>10.0	—
	SL-I	>10.0	—
	SL-C	>10.0	—
methanol extracts	R-I	1.9 ± 0.6	—
	R-C	>10.0	—
	SL-I	0.9 ± 0.4	—
	SL-C	2.1 ± 1.1	—
chicoric acid (**4a**)		—	66.7 ± 7.0
galloflavin		—	101.5 ± 10.2

^a^Values are reported as the means ± standard deviation (SD) of three or more independent experiments.

SL-C = aerial part (stem and leaves) extract from control plants; SL-I = aerial part (stem and leaves) extract from inoculated plants; R-C = root extract from control plants; R-I = root extract from inoculated plants.

Chicoric acid has been already reported as one of the main metabolite involved in the biological activity of *E*. *purpurea*^36^ and it is known for its anticancer and apoptosis inducer properties^16^ but, to the best of our knowledge, for the first time in this study it is postulated its involvement in LDH5 inhibition. Moreover, chicoric acid showed a superior activity in comparison with the LDH5 inhibitor, galloflavin^35^.

### Molecular modelling

To investigate how chicoric acid interacts with LDH5, docking calculations combined with molecular dynamics (MD) simulations and binding energy evaluations were carried out. The compound was docked into the catalytic site of LDH5 (4M49 PDB code) using AUTODOCK software^37^, and two hundred docking poses were generated and then clustered by applying a root-mean-square deviation (RMSD) of 3.0 Å. For each cluster with a population of at least five docking results, the pose associated with the best-estimated binding energy was selected as a representative binding mode. Following this approach, 12 different chicoric acid hypothetical binding modes were obtained and subjected to 20 ns of MD simulations in order to evaluate their stability. As shown in Table 2 and Supplementary Fig. S2, the analysis of the RMSD of the position of the 12 ligand poses during the MD simulations with respect to the input docking poses, highlighted that, although in each complex the ligand showed some adjustment of its binding pose due to the protein flexibility, in the case of pose 5, 8, 10, 11 and 12 the compound lost most of its starting interactions after major changes in its binding conformation and orientation. Differently, poses 1–4 and 9 showed higher stability with an average RMSD value lower than 4.0 Å.

**Table 2:** Analysis of the MD simulations of the twelve different LDH5-chicoric acid complexes.

Docking pose	Average RMSD (Å)
1	3.8
2	3.6
3	3.4
4	3.5
5	9.3
6	4.8
7	4.6
8	6.3
9	3.7
10	6.1
11	7.4
12	5.6

The average RMSD of the position of the ligand pose during the MD simulations with respect to the input docking pose is reported.

Even though the RMSD results obtained for poses 5, 8, 10, 11 and 12 strongly suggested that these binding dispositions of the chicoric acid were highly improbable, all the MD trajectories were further subjected to ligand-protein binding energy evaluation. This study was carried out by using the molecular mechanics and Poisson–Boltzmann surface area (MM-PBSA) method^38^, which has been shown to accurately estimate the ligand-receptor energy interaction^39^. In this case the MM-PBSA calculations were used for discriminating among different poses suggested by a docking calculation^34,40^. This method averages the contributions of gas phase energies, solvation free energies, and solute entropies calculated for snapshots of the complex molecule as well as the unbound components, extracted from MD trajectories, according to the procedure fully described in the Experimental Section. The MM-PBSA results (Table 3) clearly suggested that the docking pose 4 was the most reliable binding mode since it showed the best binding energy and exceed by at least 9.6 kcal/mol the interaction energies associated with the other poses.

**Table 3:** Molecular mechanics and Poisson-Boltzmann surface area (MM-PBSA) resulting values for the 12 different LDH5- chicoric acid complexes^a,b^.

Docking pose	VDW	EEL	EPB	ENP	ΔPBSA
1	−41.6	−211.6	245.1	−5.0	−13.1
2	−39.6	−208.1	239.6	−4.5	−12.6
3	−44.4	−250.7	287.8	−4.9	−12.3
4	−42.1	−239.4	262.6	−4.7	−23.6
5	−28.3	−149.4	170.3	−3.8	−11.2
6	−33.4	−245.9	271.2	−4.5	−12.6
7	−41.4	−213.1	255.2	−4.7	−4.0
8	−33.6	−190.4	215.1	−4.0	−12.9
9	−31.3	−157.0	182.2	−4.1	−10.2
10	−26.2	−126.3	146.9	−3.2	−8.8
11	−31.0	−122.0	146.4	−4.1	−10.7
12	−36.4	−211.8	239.0	−4.6	−13.8

^a^ΔPBSA is the total amount of the electrostatic (EEL), van der Waals (VDW), polar (EPB), and nonpolar (ENP) solvation free energy. ^b^Data are expressed as kcal/mol.

**Figure 3:**
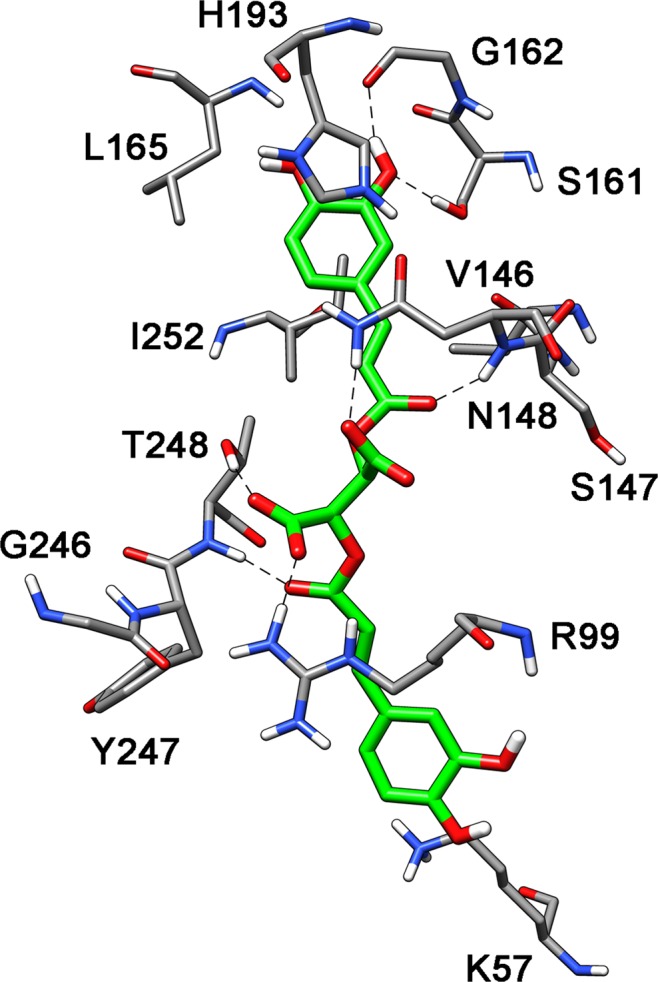
Putative binding mode of chicoric acid into *LDH*5.

Figure 3 shows the minimized average structure of the LDH5 model complexed with the chicoric acid in the suggested binding mode (cluster 04) obtained from the 20 ns of the MD simulation. One of the two 3,4-dihydroxyphenyl rings showed two H-bonds with the hydroxyl group of S161 and the oxygen backbone of G162 whereas the aromatic ring showed lipophilic interactions with L165 and I252 and a π-π interaction with H193. The carbonyl oxygen of the acrylate fragment bonded to this phenyl ring formed an H-bond with the nitrogen backbone of N148, whereas the two carboxylic groups of the ligand were stabilized by H-bonds with the side-chains of R99, N148 and T248. Finally, the second 3-(3,4-dihydroxyphenyl) acrylate fragment showed only an H-bond with the nitrogen backbone of T248 with the 3,4-dihydroxyphenyl group that did not show important interactions and was localized in the solvent exposed binding site entrance of the enzyme. Interesting, as shown in Supplementary Fig. S3, with this binding disposition, the chicoric acid fulfilled part of the binding site cavity occupied by the NADH cofactor.

Our previous results have showed that bacterial endophytes were involved in the modulation of the alkamide levels in inoculated *E*. *purpurea* plants^11^. Here we have demonstrated that the interaction between plant and endophytes influences other plant secondary metabolism patterns that is the production of pharmacological relevant phenolic compounds. Moreover, to the best of our knowledge this is the first report showing *E*. *purpurea* extracts and chicoric acid as LDH5 inhibitors. Then, our *in vitro* model on plant-bacterial interaction may lead to obtain extracts from plants enriched in compounds with therapeutic properties and represent a relevant novel approach for anticancer drug research.

## Materials and Methods

### Chemicals

HPLC grade solvents (*n*-hexane, formic acid, and methanol) were purchased from VWR (Milano, Italy). Ultrapure water (18 mΩ) was obtained with a Mill-Ω purification system (Millipore Corp., Bedford, MA, USA). Standard of chicoric acid (2,3-dicaffeoyl-l-tartaric acid, purity ≥ 95%) was obtained from Sigma (Milano, Italy).

### Bacterial and plant material

Bacterial endophytes were isolated from the aerial compartment of *E*. *purpurea* plants grown at the “Il Giardino delle Erbe”, Casola Valsenio, Italy, as previously reported^28^. “Il Giardino delle Erbe” also provided the *E*. *purpurea* seeds.

### *E. purpurea in vitro* model

Setting up of an infection model of axenic *in vitro E*. *purpurea* plants inoculated with a pool of bacterial strains isolated from the *E*. *purpurea* aerial parts was already reported^11^. Briefly, seeds were surface sterilized and germinated in Linsmaier & Skoog Medium (LS) including vitamins (Duchefa Biochemie, The Netherlands). After root (R) formation, the seedlings were maintained at 24 ± 1 °C with a photoperiod of 16 h light [fluorescent lamps, cool white, PPFD = 20.25 µmol m^−2^ s^−1^ (1500 lux)] a day for a minimum of two months. The sterility of the obtained model system was also checked. A pool of *inocula* of bacterial endophytes (Supplementary Table S1), isolated from aerial parts of *E*. *purpurea* plants, were used to infect five 2-months old *E*. *purpurea* plants, as described in Maggini *et al*.^11^. Bacterial pool was obtained joining the single *inocula* of each strain incubated for three days at 30 °C and adjusted to 8 × 10^8^ CFU/ml (OD_600_ = 1). Five plants were used as control and were inoculated with 100 μl of sterilized saline solution. After 45 days, aerial parts and roots from control and inoculated plants, were collected separately, sterilized and dried at 60 °C to be used for extract preparation. The experiment was performed in triplicate.

### Sample preparation for chemical analysis

Dried and powdered aerial parts and roots of *E*. *purpurea*, both non-inoculated (1.26 g aerial parts and 0.28 g roots) and endophyte-inoculated (0.92 g aerial parts and 0.24 g roots), were subjected to extraction at room temperature first with a nonpolar solvent (*n*-hexane) and successively with methanol (1.0 g of dried drug in 30 ml); each solvent was renewed every day for three times and the obtained extracts were recovered by filtration using filter paper. Finally, the solvents were removed under vacuum, obtaining 17.4 mg and 370.9 mg from non-inoculated aerial parts and 7.8 mg and 58.9 mg from non-inoculated roots, and 11.2 mg and 282.9 mg from endophyte-inoculated aerial parts and 2.4 mg and 61.6 mg from endophyte-inoculated roots of *n*-hexane and methanol residues, respectively. The *n*-hexane portions were analyzed for their alkamide content as previously reported^11^. Since methanol extracts from aerial parts were rich in chlorophylls, they were previously purified by Solid Phase Extraction (SPE) using cartridges containing C-18 reverse phase silica gel: the extracts were dissolved in methanol and centrifuged, thereafter the supernatant solutions were subjected to SPE eluting with methanol-water solution (9:1). The recovered eluates were dried under vacuum obtaining dried residues of 165.9 mg (control) and 150.7 mg (endophyte-inoculated). Finally, the obtained residues from aerial parts together with methanol extracts obtained from roots were dissolved in methanol at a concentration of 2.5 mg/ml, centrifuged and injected in triplicate (20 μl) into the HPLC-PDA-MS system for quali-quantitative investigation of their phenolic content.

### HPLC-PDA/UV-ESI-MS/MS analyses of *E*. *purpurea* phenolic content

Quali-quantitative HPLC-PDA/UV-ESI-MS/MS analyses of *E*. *purpurea* roots and aerial parts, focused on phenolic constituents, were carried out using a Thermo Scientific HPLC system (San Jose, CA, USA) composed by a Surveyor LC pump and a Surveyor autosampler, coupled with a Surveyor PDA/UVvis detector and a LCQ Advantage ion trap mass spectrometer equipped with an ESI source. Data were acquired with with Xcalibur 3.1 software. Chromatographic analyses were achieved on a Synergi Fusion-RP column (4.6 × 250 mm, 4 μm, Phenomenex, Bologna, Italy) using a mobile phase composed by methanol (solvent A) and a 0.1% formic acid aqueous solution (solvent B) and developing a linear gradient of increasing methanol from 5 to 60% within 60 minutes; thus, the column was successively washed for 15 minutes with methanol and equilibrated with 5% methanol for 10 minutes. Samples were injected in a volume of 20 μl, at a flow rate of 0.8 ml/minute with a splitting system of 2:8 to MS detector (160 μl/minute) and PDA detector (640 μl/minute), respectively. Mass spectra were acquired with an ESI interface operating in negative ion mode and N_2_ was used as the sheath and auxiliary gas. The thune method for ionization was as follows: spray voltage, 4.50 kV; capillary voltage, −11.00 V; tube lens offset, −45.0 V; capillary temperature, 270 °C; sheath gas flow rate, 60.00 arbitrary units; auxiliary gas flow rate, 3.00 arbitrary units. The normalized collision energy in the MS/MS experiments was 35%. MS data were recorded over scan range of *m/z* 150–1500, while PDA/UV data were registered over 200–600 nm range, with preferential channels 254, 280, and 330 nm as the detection wavelengths. The amount of main phenolic components of methanol axenic and endophyte-inoculated root and aerial part extracts was determined using a calibration curve constructed by using the chicoric acid as external standard in a concentration range 0.005–1.0 mg/ml and methanol as solvent. Methanolic standard solution at four different concentrations (0.005, 0.01, 0.1 and 0.25 mg/ml) were analysed by triplicate injections and plotted versus the area derived from integrated PDA/UV peaks registered at 330 nm to generate the calibration curve. A linear simple correlation was used to analyze the relation between variables. For the linear regression of the standard, *R*^2^ was 0.9726. The amounts of detected phenols (expressed as mg/1 g of fresh plant material) were obtained by using a GraphPad Software Prism 6.0.

### Lactate dehydrogenase (LDH)assay

The LDH inhibition activities of extracts and pure chicoric acid were tested against purified human lactate dehydrogenase isoform 5 (purchased by Lee Biosolution, Inc.). The reaction was run in the “forward” direction (from pyruvate to lactate) by monitoring the quantity of NADH at 340 nm which was consumed after 15 minutes incubation. Assays were performed in 96 well plates, and in each well was present a final volume of 200 µl. Pyruvate (200 μM) and NADH (40 μM) were dissolved in phosphate buffer (100 mM, pH = 7.4), containing BSA (0.1 mg/ml). DMSO stock solutions of the tested extracts and of compound **4a** (final concentrations: for extracts 5 mg/ml, for **4a** 500 µM) were serially diluted to obtain seven different concentrations to obtain the concentration-response curve. In each plate, maximum controls are included, that are wells corresponding to the maximum enzyme activity. The “maximum” wells do not contain compounds, which are substituted by DMSO, but they contain LDH enzyme, therefore the enzyme has the maximum activity since it is not inhibited by compounds. Minimum controls are also present in each plate: in these wells the enzyme is not present and it is substituted by buffer, therefore these wells correspond to no enzyme activity, and for this reason they are defined as “minimum” controls. The final readings were performed by using a Victor X3 microplate reader (PerkinElmer) and IC_50_ values were calculated from experimental data with the sigmoidal dose-response fitting of GraphPad Prism software.

### Docking calculations

The crystal structure of the LDH5 protein (4M49 PDB code) was taken from the Protein Data Bank^41^. After adding hydrogen atoms, the protein was minimized using Amber16 software and the ff14SB force field at 300 K. The complex was placed in a rectangular parallelepiped water box, an explicit solvent model for water, TIP3P, was used and the complex was solvated with a 10 Å water cap. Sodium ions were added as counterions to neutralize the system. Two steps of minimization were then carried out; in the first stage, we kept the protein fixed with a position restraint of 500 kcal/molÅ^2^ and we solely minimized the positions of the water molecules. In the second stage, we minimized the entire system through 5000 steps of steepest descent followed by conjugate gradient (CG) until a convergence of 0.05 kcal/Åmol. The ligand was built using Maestro and was minimized by means of Macromodel in a water environment using the CG method until a convergence value of 0.05 kcal/Åmol, using the MMFFs force field and a distance-dependent dielectric constant of 1.0. AUTODOCK Tools^37^, was used to define the torsion angles in the ligands, to add the solvent model and to assign partial atomic charges to the ligand and the protein.

### Molecular dynamic (MD) simulations

All simulations were performed using AMBER, version 16^42^. Molecular dynamic (MD) simulations were carried out using the ff14SB force field at 300 K. The complex was placed in a rectangular parallelepiped water box. An explicit solvent model for water, TIP3P, was used, and the complexes were solvated with a 20 Å water cap. Chlorine ions were added as counterions to neutralize the system. Prior to MD simulations, two steps of minimization were carried out using the same procedure described above. Particle mesh Ewald (PME) electrostatics and periodic boundary conditions were used in the simulation. The MD trajectory was run using the minimized structure as the starting conformation. The time step of the simulations was 2.0 fs with a cutoff of 10 Å for the nonbonded interaction, and SHAKE was employed to keep all bonds involving hydrogen atoms rigid. Constant-volume periodic boundary MD was carried out for 0.5 ns, during which the temperature was raised from 0 to 300 K. Then 19.5 ns of constant pressure periodic boundary MD was carried out at 300 K using the Langevin thermostat to maintain constant the temperature of our system. All the α carbons of the protein were blocked with a harmonic force constant of 10 kcal/mol•Å^2^ for the first 3.5 ns. General Amber force field (GAFF) parameters were assigned to the ligand, while partial charges were calculated using the AM1-BCC method as implemented in the Antechamber suite of AMBER 16. The final structures of the complexes were obtained as the average of the last 16.5 ns of MD minimized by the CG method until a convergence of 0.05 kcal/mol•Å^2^. The average structures were obtained using the Cpptraj program implemented in AMBER 16.

### Binding energy evaluation

The evaluation of the binding energy associated to the different ligand-protein complexes analyzed through MD simulations was carried out using AMBER 16 as already reported^43^. The trajectories relative to the last 16.5 ns of each simulation were extracted and used for the calculation, for a total of 165 snapshots (at time intervals of 100 ps).

### Statistical analyses

Means and standard deviations of the bacterial TVC data of the three biological replicates were estimated and compared by one-way analysis of variance between root and aerial part samples^11^. To evaluate whether the level estimations of the phenolic compounds (mg in 1 g of plant fresh weight) were useful in reflecting the chemical relationships between root and aerial part samples (controls and inoculated), a PCA was performed^44^. One-way analysis of variance followed by Tukey test was used to compare phenolic amount values between control and inoculated plants and to identify the compounds mainly responsible of the differences between the samples. *P* < 0.05 was considered significant (*P < 0.05, **P < 0.01, ***P < 0.001)^7^. Error bars are shown as standard error of the mean (Sem). The analyses were performed by using the modules present in the PAST program, version 3.15^45^.

## Supplementary information

Supplementary Material

## Data Availability

All data generated or analysed during this study are included in this published article (and its Supplementary Information files) or available from the corresponding author on reasonable request.
