# Supplementary material for: The influence of Echinacea purpurea leaf microbiota on chicoric acid level
Source: Sci Rep. 2019 Jul 26;9:10897. doi: 10.1038/s41598-019-47329-8 (PMC6659708; doi:10.1038/s41598-019-47329-8)

The influence of *Echinacea purpurea* leaf microbiota on chicoric acid level

Valentina Maggini[1,2,3*], Marinella De Leo[4,5], Carlotta Granchi[4], Tiziano Tuccinardi[4], Alessio Mengoni[1], Eugenia Rosaria Gallo[2,3], Sauro Biffi[6], Renato Fani[1], Luisa Pistelli[4,5], Fabio Firenzuoli[3] and Patrizia Bogani[1]

[1] Department of Biology, University of Florence, Via Madonna del Piano 6, 50019 Sesto Fiorentino, Italy

[2] Department of Experimental and Clinical Medicine, University of Florence, Largo Brambilla 3, 50134 Florence, Italy

[3] Referring Center for Phytotherapy, Tuscany Region, Careggi University Hospital, Largo Brambilla 3, 50134 Florence, Italy

[4] Department of Pharmacy, University of Pisa, Via Bonanno 6 and 33, 56126 Pisa, Italy

[5] Research Centre for Nutraceutical and Healthy Foods "NUTRAFOOD", University of Pisa, via del Borghetto 80, 56124 Pisa, Italy

[6] Botanical Garden Casola Valsenio, Via del Corso 6, 48010 Ravenna, Italy

Valentina Maggini and Marinella De Leo contributed equally.

Fabio Firenzuoli and Patrizia Bogani jointly supervised this work.

Corresponding Author

* Dr Valentina Maggini, Laboratory of Plant Genetics, Dept. of Biology, University of Florence, Via Madonna del Piano 6, 50019 Sesto Fiorentino, Italy Tel +39 0554574731, Email valentina.maggini@unifi.it

Supplementary Table S1. The thirty-seven bacterial strains from *E. purpurea* used in this work (Maggini et al., 2017).

Strain code	Genus	GenBank accession of partial 16S rRNA gene sequence
EpSL1	*Curtobacterium* sp.	KJ642423
EpSL2	*Curtobacterium* sp.	KJ642424
EpSL4	*Microbacterium* sp.	KJ642438
EpSL5	*Bacillus* sp.	KJ642422
EpSL16	*Arthrobacter* sp.	KJ642432
EpSL17	*Staphylococcus* sp.	KJ642469
EpSL18	*Arthrobacter* sp.	KJ642419
EpSL20	*Pseudomonas* sp.	KJ642444
EpSL22	*Staphylococcus* sp.	KJ642476
EpSL25	*Pseudomonas* sp.	KJ642442
EpSL27	*Arthrobacter* sp.	KJ642420
EpSL31	*Rhodobacter* sp.	KJ642453
EpSL32	*Sphingomonas* sp.	KJ642455
EpSL34	*Staphylococcus* sp.	KJ642465
EpSL35	*Arthrobacter* sp.	KJ642421
EpSL37	*Pseudomonas* sp.	KJ642443
EpSL39	*Pseudomonas* sp.	KJ642336
EpSL40	*Staphylococcus* sp.	KJ642466
EpSL43	*Pseudomonas* sp.	KJ642443
EpSL50	*Sphingomonas* sp.	KJ642457
EpSL54	*Frigoribacterium* sp.	KJ642425
EpSL59	*Frigoribacterium* sp.	KJ642426
EpSL62	*Staphylococcus* sp.	KJ642472
EpSL64	*Frigoribacterium* sp.	KJ642427
EpSL65	*Microbacterium* sp.	KJ642440
EpSL70	*Sphingomonas* sp.	KJ642460
EpSL80	*Frigoribacterium* sp.	KJ642428
EpSL81	*Agrococcus* sp.	KJ642418
EpSL82	*Methylobacterium* sp.	KJ642437
EpSL83	*Sphingomonas* sp.	KJ642462
EpSL84	*Frigoribacterium* sp.	KJ642430
EpSL87	*Kineococcus* sp.	KJ778698
EpSL89	*Staphylococcus* sp.	KJ642473
EpSL91	*Frigoribacterium* sp.	KJ642429
EpSL95	*Staphylococcus* sp.	KJ642470
EpSL96	*Staphylococcus* sp.	KJ642474
EpSL102	*Staphylococcus* sp.	KJ642464

Supplementary Table S2. Spectral (UV and ESI-MS/MS) and chromatographic data (retention time, t_R) of phenols (**1-6**) detected in both control and endophyte-inoculated root (R) and aerial part (stem and leaves; SL) methanol extracts of *Echinacea purpurea*. Compounds correspond with peaks in Fig. 1

Peak	Compound	t_R (min)	λ_{max} (nm)	M	$[M-H]^-$	MS/MS base peak (*m/z*)	MS/MS ions (*m/z*)	Organ
Phenolic acids								
**1**	Dihydroxybenzoic acid hexoside	17.3	240, 315	316	315	153	271, 109	R, SL
**2**	Caftaric acid	24.0	255, 290, 330	312	311	179	293, 149, 135	R, SL
**3**	Chlorogenic acid	31.0	245, 330	354	353	191	179, 161, 135	R
**4a/4b**	Chicoric acid	44.8/45.5	250, 330	474	473	311	341, 293, 179, 149	R, SL
**5**	Dicaffeolylquinic acid	46.3	255, 285, 330	516	515	353	335, 275, 191, 179	R
Flavonoids								
**6**	Rutin	49.2	260, 355	610	609	301	591, 463, 271, 255	R

Supplementary Table S3. Average, standard deviation (SD) and comparison of the amounts of phenolic compounds identified in the extracts from root and aerial part samples **(**SL-C: aerial part (stem and leaves) extract from control plants; SL-I: aerial part (stem and leaves) extract from inoculated plants; R-C: root extract from control plants; R-I: root extract from inoculated plants). Pairwise comparison (Tukey HSD) was performed between inoculated samples and the relative controls: different letters in the same column indicate significant differences ($P < 0.05$). Compound numbers correspond with peak numbers in Fig. 1

	Phenolic compound amounts (mg/g of fresh weight) mean ± SD				
	**2**	**3**	**4**	**5**	**Phenolic Content**
R-C	0.089 ± 0.005	0.089 ± 0.002	0.289 ± 0.015	0.016 ± 0.001	0.484 ± 0.117
R-I	0.039 ± 0.001	0.034 ± 0.001	0.177 ± 0.010[a]	0.020 ± 0.001	0.270 ± 0.073
SL-C	0.129 ± 0.005	nd	0.336 ± 0.012	nd	0.466 ± 0.159
SL-I	0.129 ± 0.005	nd	0.490 ± 0.019[b]	nd	0.619 ± 0.487

nd = not detected

Supplementary Fig. S1. Phenolic compound amounts differentiate the *E. purpurea* extracts. Biplot from Principal Component Analysis was carried out with phenolic compound estimations of the four different extracts [SL-C: aerial part (stem and leaves) extract from control plants; SL-I: aerial part (stem and leaves) extract from inoculated plants; R-C: root extract from control plants; R-I: root extract from inoculated plants]

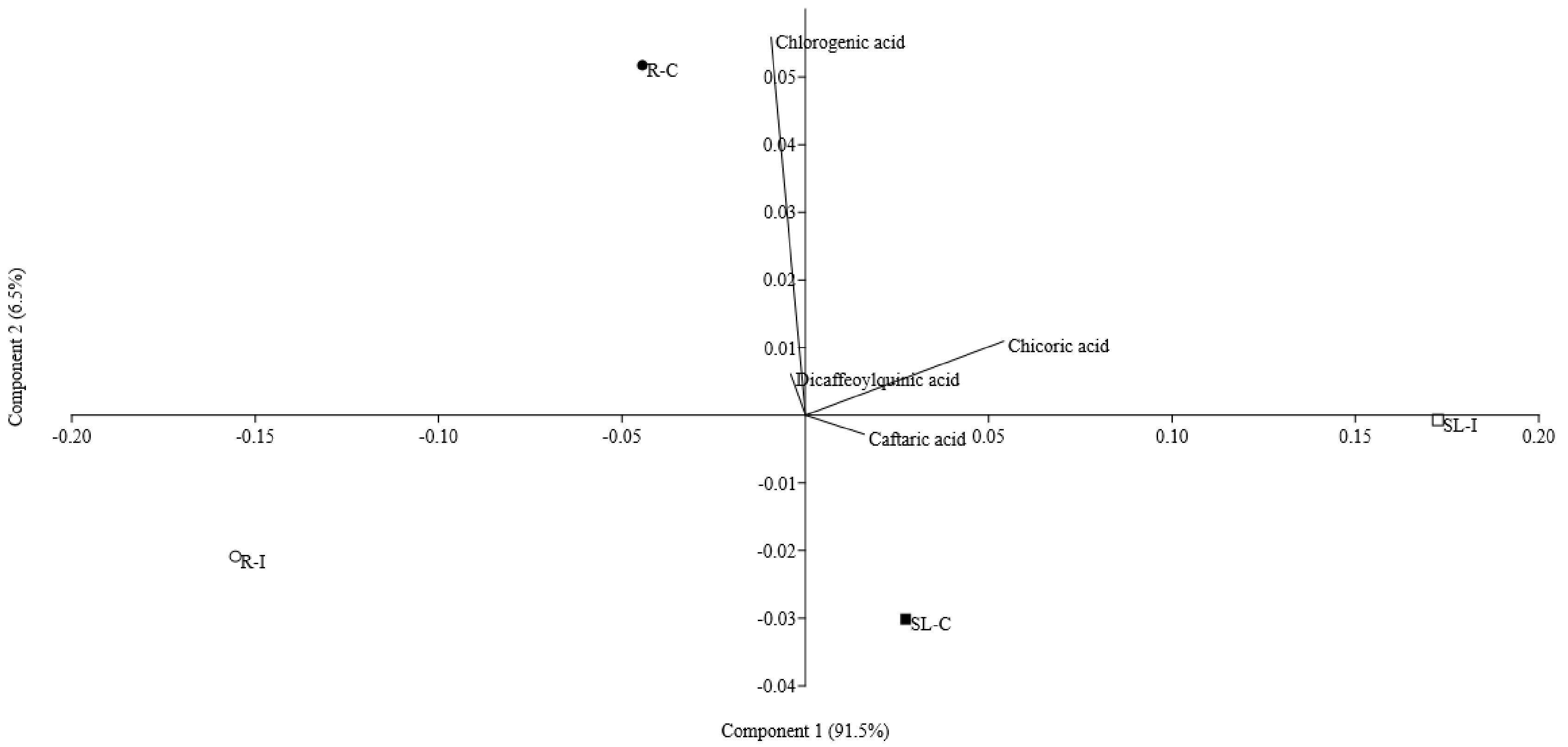

Supplementary Fig. S2. Analysis of the MD simulations of the different *h*LDH5– chicoric acid complexes. The plot shows the RMSD of the position of the ligand with respect to its initial docking pose.

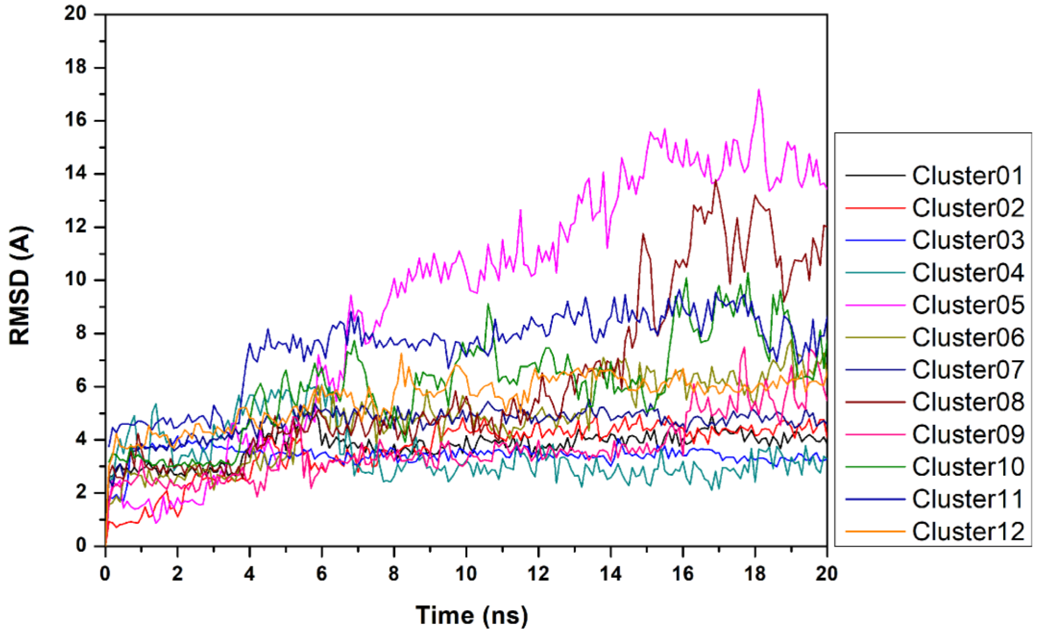

Supplementary Fig. S3. Superimposition between the putative binding mode of chicoric acid (green) and NADH (yellow) into *h*LDH5.

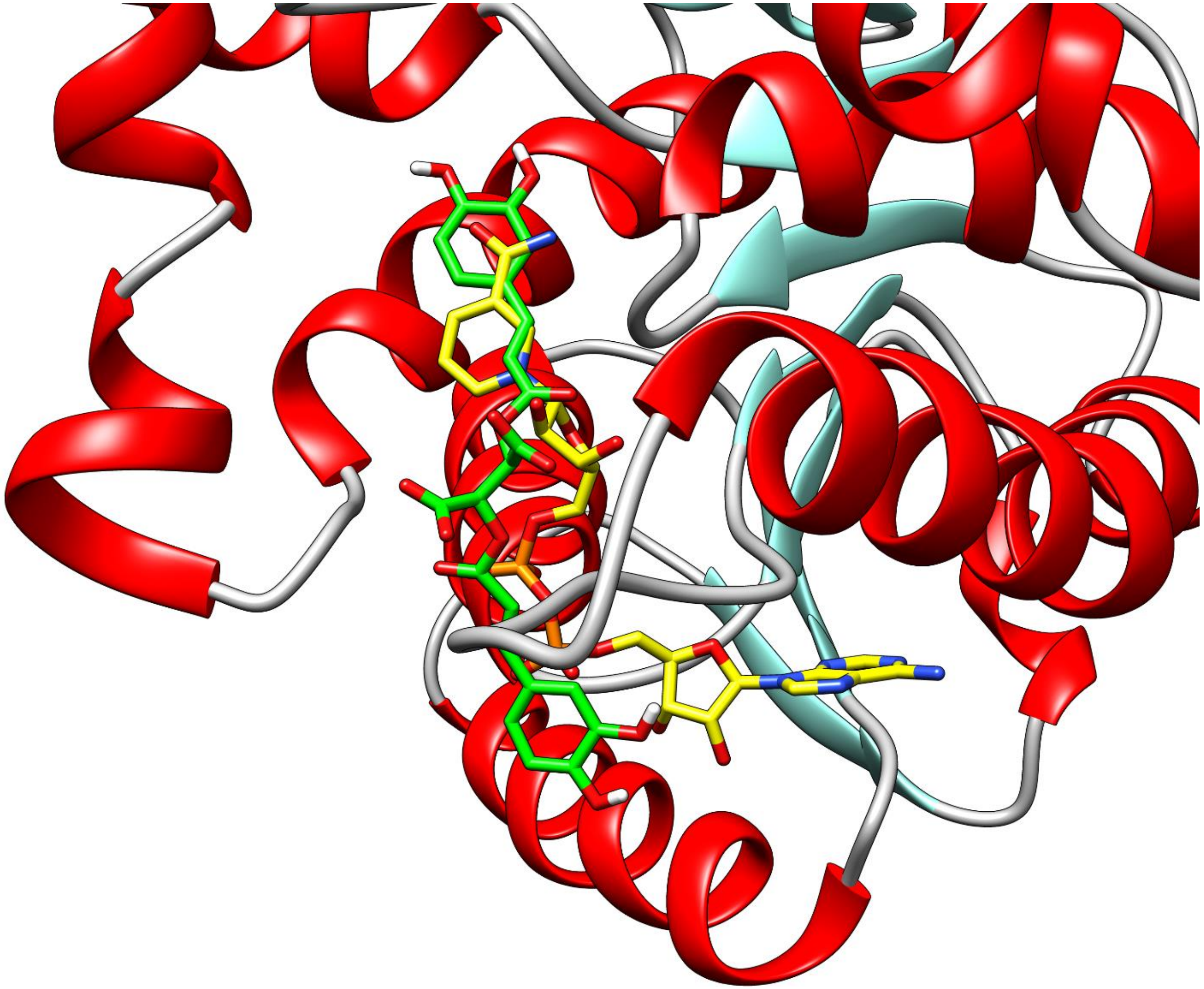

Supplement: Supplementary file 1 — Supplementary Material [file 41598_2019_47329_MOESM1_ESM.pdf]
